# Repurposing rupatadine to attenuate ovarian ischemia reperfusion in rats through modulation of PAF/NF-κB/TNF-α/IL-1β; HIF-1α/VEGF/Caspase 3 signaling pathways

**DOI:** 10.1007/s00210-025-04567-0

**Published:** 2025-09-05

**Authors:** Hanaa Mohamed Khalaf, Sara M. Ahmed, Alyaa Abdelfattah Abdelmonaem, Rabeh Khairy Saleh, Abdelaleem Abdelnour Mohamed, AbdelHamid Sayed AboBakr Ali, Mohamed Adel, Heba Reda Mohamed, Heba S. Kamel, Walaa Yehia Abdelzaher

**Affiliations:** 1 Department of Medical Pharmacology, Faculty of Medicine, Minia University, Minia, 61519 Egypt; 2 Department of Pathology, Faculty of Medicine, Minia University, Minia, 61519 Egypt; 3 Department of Medical Physiology, Faculty of Medicine, Minia University, Minia, 61519 Egypt; 4 Department of Anatomy, Faculty of Medicine, Minia University, Minia, 61519 Egypt; 5 Department of Obstetrics & Gynecology, Faculty of Medicine, Minia University, Minia, 61519 Egypt; 6 Department of Medical Biochemistry, Faculty of Medicine, Minia University, Minia, 61519 Egypt; 7 Faculty of Nursing, Lotus University, Minia, 61768 Egypt

**Keywords:** Ovarian ischemia reperfusion, Rupatadine, PAF, VEGF, HIF-1α

## Abstract

The aim of the current study is to identify the possible protective effect of rupatadine (RUP) on ovarian ischemia reperfusion (OIR) in rats. RUP was administered in the presence and absence of OIR. Thirty-two adult Wistar albino female rats were randomly arranged into four groups: Sham, RUP (6 mg/kg/day) for 14 days, OIR and OIR + RUP groups.The results demonstrated that OIR significantly lowered serum anti-mullerian hormone level and ovarian total antioxidant capacity. Besides, a significant elevation in serum follicle stimulating hormone, lutenizing hormone, ovarian malondialdehyde level, hypoxia-inducible factor-1(HIF-1α), platelet activating factor (PAF), tumor necrosis factor-alpha (TNF-α), nuclear factor kappa-light-chain-enhancer of activated B cells (NF-κB) and interleukin 1beta (IL-1β) levels along with an evident increase in ovarian vascular endothelial growth factor (VEGF) and caspase-3 immunoexpression. While, OIR + RUP pretreated group showed a reversal in OIR damaging effects in a significant manner in all the aforementioned parameters. Based on these findings; RUP has powerful anti-IR actions by lowering oxidative stress, inflammation, and apoptosis via modulation of the PAF/ NF-κB/TNF-α/ IL-1β; HIF-1α/ VEGF / Caspase 3 signaling pathways.

**Graphical abstract:**

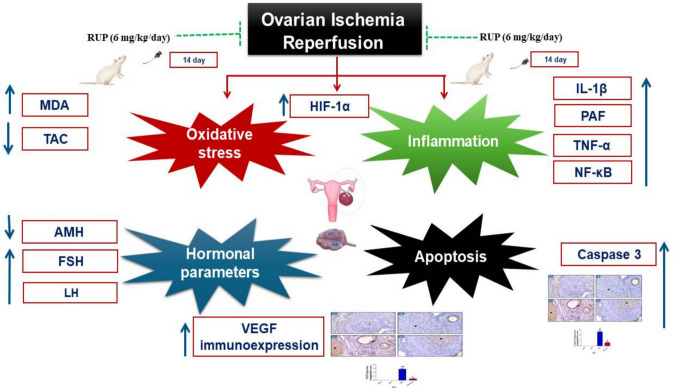

## Introduction

Ischemia (I) is the decrease or interruption of blood flow to an organ as a result of blockage or mechanical factors. Reperfusion, on the other hand, refers to the return of blood flow (R). Although it can cause reperfusion injury, reperfusion aids in returning ischemic tissue to normal function. About 30% of ovarian torsion instances occur in people under the age of twenty. It is a common gynecological emergency, especially in young women of reproductive age (Öztürk et al. 2025). Blood flow disruptions brought on by the ovary’s rotation along its axis can lead to serious side effects including bleeding, adhesions, thrombophlebitis, sepsis, and even death. Infertility, necrosis, and other potentially fatal consequences can result from ovarian torsion, which dramatically raises morbidity and mortality if it is not identified or treated quickly. Hypoxic tissues are oxygenated through detorsion, which produces oxygen radicals and damages the tissue. The oxidative-antioxidative balance is upset in favor of oxidative stress by increased generation of oxygen radicals and consumption of antioxidants. Numerous substances were experimentally employed to treat ovarian IR (OIR) injury in order to support the antioxidant cycle (Ulusoy Tangul et al. 2025).

Numerous drugs may be useful because reactive oxygen species (ROS) generation and enhanced cellular apoptosis are the main causes of ovarian damage. Certain medications, such as bromelain, selenium, carotenoids, vitamin C, protein, erythropoietin, vardenafil, and curcumin, may be able to mitigate cellular damage after OIR, according to both experimental and clinical studies. These treatments could serve as a strong foundation for an adjuvant-restoring therapy that aims to improve ovarian function following torsion (Kirmizi et al. 2021; Yilmaz et al. 2023; Ulusoy Tangul et al. 2025). There is currently no medication that is clinically effective for reducing OIR damage.

Rupatadine (RUP) is a second-generation antihistamine that has been licensed for the treatment of allergic disorders and chronic urticaria. It has a long-acting blocking effect on both the platelet-activating factor (PAF) receptor and the histamine-1 (H1) receptor. Numerous studies have demonstrated that RUP suppresses the release of pro-inflammatory cytokines, including interleukin-6 (IL-6) and tumor necrosis factor-alpha (TNF-α) caused by PAF, hence exerting strong antioxidant and anti-inflammatory effects (Khalaf et al. 2022). Additionally, RUP can inhibit the release of pro-inflammatory cytokines such as IL-3, IL-6, IL-8, and TNF-α as well as histamine from mast cells because it is an H1-receptor antagonist. RUP has been shown to protect against testicular ischemia/reperfusion injury and to prevent mast cell-dependent leukocyte recruitment during cardiac ischemia/reperfusion (Abdel-Aziz et al. 2024; Gökçek et al. 2024). In models of isoproterenol-induced heart failure and bleomycin- and silica-induced pulmonary fibrosis, RUP has been shown to aid in the resolution of inflammation and fibrosis (Ahmed et al. 2021). Furthermore, via altering the PAF/IL-6/vascular endothelial growth factor (VEGF) signaling pathway, RUP has been demonstrated to prevent ulcerative colitis in rats (Ibrahim et al. 2022).

Based on these results, the current study intends to examine the modulatory effects of RUP on OIR injury in rats and the possible involvement of PAF/HIF-1α/VEGF/Caspase-3 signaling and nuclear factor kappa-light-chain-enhancer of activated B cells (NF-κB)/TNF-α/IL-1β.

## Materials and methods

### Chemicals

Sigma-Aldrich Co. (USA) was the supplier of rupatadine (Rup). The enzyme-linked immunosorbent assay (ELISA) kits for TNF-α, IL-1β, PAF, and HIF-1α (catalog numbers: E-EL-R2856, E-EL-R0012, EK720826, and DL-HIF1a-Ra) were acquired from Elabscience Co. (USA), AFG Scientific Co. (USA), and Di Develop Co., respectively. Wuhan Fine Biological Technology Co., Wuhan, Hubei, China, provided the follicle stimulating hormone (FSH) ELISA Kit (Catalog number: ER0960). The ELISA kits for luteinizing hormone (LH) (Catalog number: CSB-E12654r) and anti-mullerian hormone (AMH) (Catalog number: E-EL-R0640) were acquired from Cusabio Co. (USA) and Elabscience Co. (USA), respectively.

### Animals

ARRIVE standards and the U.K. Animals Act, 1986, were followed in the procedures concerning the care of animals and the laboratory environment. Minia protocol clearance number (1296:10/2024) was received from our university’s local ethics committee and authorized by the Faculty of Medicine board. For this investigation, a total of 32 adult female Wistar albino rats weighing 200–250 g were provided by the National Research Center in Cairo, Egypt. The rats were allowed to freely drink tap water and eat a standard rat chow diet for around two weeks before the current study started so they could get used to the lab setting. The experiment was conducted with a 12-h light/dark cycle at 24 °C ± 2°C.

### Experimental design

Four groups of eight rats each were randomly selected from among the animals:

- Group 1: Sham group: rats received oral carboxymethylcellulose (CMC) as a vehicle for 14 days and underwent all surgical procedures except OIR.
- Group 2: RUP group: Rats were introduced to all surgical procedures without OIR after receiving RUP 6 mg/kg orally once daily suspended in CMC for 14 days (Hafez et al. 2020).
- Group 3: OIR: (ovarian ischemia reperfusion + Vehicle): Rats received oral CMC (vehicle) for 14 days before OIR induction. This group served to control for the effects of the vehicle and the surgical procedure.
- Group 4: OIR + RUP group: rats were given oral RUP at a dose of 6 mg/kg/day for 14 days before OIR induction.

#### OIR surgical procedures

All surgical operations were performed utilizing IM ketamine anesthesia (50 mg/kg) in sterile settings in suitable lab settings. After shaving the abdomen region and cleaning it with 2% iodine alcohol, rats were put in a supine position and had a laparotomy through a lower longitudinal abdominal incision (2.5 cm). A vascular clamp was placed on the inferior portion of the ovaries to perform bilateral ovarian ischemia for three hours. After that, 3/0 nylon sutures were used to close the abdominal wall incision. Three hours after ischemia, the abdominal wall was reopened, and the clamp was taken off to permit another three hours of normal reperfusion. Ekinci Akdemir et al. (2021) reported that the abdomen was once more closed; after clamping and ischemia induction, the abdomen was temporarily closed and then reopened for reperfusion. Following the procedure, the abdominal wall was re-closed with sutures. This step was indeed done to restore anatomical integrity, decrease the risk of infection or dehydration, and ensure the physiological stability of the animal during the reperfusion period.

#### Sample collection and storage

Rats were weighed and sacrificed following the surgical operation. Blood samples were drawn from the abdominal aorta and centrifuged for 10 min at 5,000 rpm to extract serum, which was then kept at −80 °C until it was needed for the biochemical analysis.

Following dissection, the ovaries were quickly removed, cleansed of blood with 0.9% saline, and weighed. For histological and immunohistochemical analysis, one ovary from each rat’s side was immersed in 10% formalin, whereas the other one was kept for RT-PCR and chemical analysis at −80 °C. After homogenizing 0.1 g of the ovary in ice-cold phosphate-buffered saline at pH 7.4 (20% w/v), it was centrifuged for 15 min at 5,000 rpm. Until they were utilized for various parameter measurements, the supernatants were stored at −80 °C.

### Biochemical analysis

#### Hormonal assay

ELISA kits were used to measure the serum levels of FSH, LH, and AMH in accordance with the manufacturer’s instructions.

#### Evaluation of ovarian oxidative stress parameters

As directed by the manufacturer, colorimetric commercial kits were used to measure malondialdehyde (MDA) (Biodiagnostic, Egypt, Catalog No: MD 25 29) and total antioxidant capacity (TAC) ((Biodiagnostic, Egypt, Catalog No: TA 25 13).

#### Assessment of anti-inflammatory parameters

Following the manufacturer’s instructions, ELISA kits were used to quantify the levels of TNF-α, Interleukin-1β (IL-1β), platelets activating factor (PAF), and Hypoxia-Inducible Factor-1 Alpha (HIF-1α) in ovarian tissue homogenates.

#### Real-time reverse transcription polymerase chain reaction (RT-PCR)

100 μg of rat ovarian tissue was treated with TRI REAGENT solution (Ambion, Warrington, UK, Cat no: TR 118) to extract total RNA. As directed by the kits (GoTaq® 1-Step RT-qPCR syber green + ROX Vial Cat no: A6020), 4 μg of total RNA was used per 20-μL reaction using the following primers in a heat cycler (Applied Biosystems GeneAmp® 5700 fast, Cambridge LTD., UK). The RNA’s purity and concentration were assessed using the Nanodrop. The following was the PCR amplification cycle methodology: After initial denaturation at 95 °C for 10 min, 40 cycles of amplification were performed, which included denaturation at 95 °C for 10 s, annealing at 60 °C for 30 s, and extension at 72 °C for 30 s. For each sample, gene expression was expressed relative to that of the sham group. Relative expressions of NF-κB gene was measured using the comparative threshold cycle method (Ct) (1).all values were standardized to the B-actin gene (Lefever et al. 2009).

The used primers were:

- B-actin gene:
  - F: 5′- TAC AGC TTC ACC ACC ACA GC-3′
  - R: 5′- GGA ACC GCT CAT TGC CGA TA-3′
- NF-κB gene:
  - F: 5′- ATC CAT GGA AGC AAG TCG AT-3′
  - R: 5′- CCT TTT GCT GTG ATC TTC CT-3′

### Histopathological assessment and scoring

The ovarian tissues were treated and embedded in paraffin wax before being fixed in 10% neutral buffered formalin for light microscopy. Hematoxylin and eosin (H&E) staining was applied to specimens embedded in paraffin wax after they were cut into 4 μm thick sections and placed on slides (Bancroft and Layton 2013). A light microscope was used to observe the tissue sections, and an Olympus BX50 camera attached to the microscope was used to capture pictures.

The results of the microscopic evaluation were scored according to Saygin et al. (2011) grading into four grades semi-quantitatively; grade 1 = absent (no finding is detected in the fields), grade 2 = mild changes (There was evidence of any finding in < 25% of the fields), grade 3 = moderate changes (There was evidence of any finding in 25–50% of the fields), grade 4 = severe changes (There was evidence of any finding in > 50% of the fields). The parameters for tissue damage included hemorrhage in the corpus luteum, vascular congestion in the ovarian stroma and mononuclear cellular infiltrate. Total scores were calculated according to these parameters (Saygin et al. 2011; Melekoglu et al. 2018)**.**

### Immunohistochemical study

The primary antibodies against caspase 3 and VEGF were kept in a moist box at 4 °C for 12 h after the slides were blocked with 3% hydrogen peroxide at room temperature for 10 min. After three PBS (phosphate-buffered saline) washes, the cells were treated for ten minutes at room temperature with horseradish peroxidase. Positive staining for VEGF and caspase 3 was observed. One control tissue was prepared for each run by removing the particular main antibody and substituting PBS for it throughout the staining process. To verify the absence of secondary antibody cross-reactivity and other non-target cell components, the negative control sections were inspected for the presence of particular staining (Huang et al. 2012).

On a semiquantitative scale, ovarian cells stained with caspase 3 were scored as positive (+) if they stained less than 33%, 33–66%, or more than 66% of the ovarian slice, respectively, and 0 denoted no staining. By methodically scoring at least 100 ovarian cells each field in 10 fields of tissue slices at a × 100 magnification, the number of caspase-3-positive cells was determined (Ergenoglu et al. 2013).At 400 × magnification, VEGF positivity was assessed in five randomly chosen regions. The staining scores were zero, weak, dense, and intense (0, + 1, + 2, + 3), respectively (Tanriverdi et al. 2016; Ersoy Canillioglu et al. 2020).

### Analysis of statistics

The mean ± standard error of the mean (SEM) was used to express the statistics from the current investigation. Post-hoc test (Tukey’s multiple comparisons test) and a one-way ANOVA were used to examine the experiment’s findings. Version 5 of GraphPad Prism was utilized. A p-value of less than 0.05 was deemed significant.

## Results

### Effect of RUP on hormonal parameters in female rats with OIR

There was a significant rise in the serum levels of FSH and LH in the OIR group along with a significant reduction in the serum levels of AMH when compared to both the sham and RUP groups. On the other hand, pretreatment with RUP before OIR led to a significant decrease in serum levels of FSH and LH and a significant increase in the level of AMH when compared to the OIR group (Table 1).

**Table 1:** Effect of RUP on hormonal parameters in female rats with OIR

Group	AMH (ng/ml)	FSH (IU/L)	LH (IU/L)
Sham	1.12 ± 0.03	5.30 ± 0.27	1.14 ± 0.04
RUP	1.05 ± 0.04	5.56 ± 0.18	1.19 ± 0.04
OIR	0.18 ± 0.01^ab^	10.57 ± 0.14^ab^	4.61 ± 0.31^ab^
OIR + RUP	1.023 ± 0.05^c^	6.94 ± 0.19^abc^	1.46 ± 0.08^c^

*RUP* rupatadine, *OIR* ovarian ischemia reperfusion, *FSH* follicle-stimulating hormone, *LH* luteinizing hormone, *AMH* anti-mullerian hormone

Data are expressed as mean ± SEM. (*n* = 8 rats/group). Statistical analysis was performed using one-way ANOVA followed by Tukey’s multiple comparisons post hoc test (values were considered significantly different when *P* < 0.05 (95% CI). ^a^Significant difference from sham group, ^b^significant difference from RUP group, ^c^significant difference from OIR group

### Effect of RUP on ovarian oxidative stress parameters in female rats with OIR

In contrast to the sham and RUP groups, OIR considerably decreased the ovarian TAC concentration while significantly increasing the ovarian MDA, as indicated in Table 2. When compared to the OIR group, the administration of RUP prior to IR dramatically reduced the observed IR-induced alterations in ovarian markers of oxidative stress.

**Table 2:** Effect of RUP on ovarian oxidative stress parameters in female rats with OIR

Group	OvarianTAC (mmol/L)	Ovarian MDA (nmol/g tissue)
Sham	1.91 ± 0.01	11.48 ± 0.39
RUP	1.79 ± 0.04	11.31 ± 0.38
OIR	0.57 ± 0.02^ab^	37.10 ± 1.20^ab^
OIR + RUP	1.59 ± 0.05^abc^	21.30 ± 0.64^abc^

*RUP* rupatadine, *OIR* ovarian ischemia reperfusion, *TAC* total antioxidant capacity, *MDA* malondialdehyde

Data are expressed as mean ± SEM. (*n* = 8 rats/group). Statistical analysis was performed using one-way ANOVA followed by Tukey’s multiple comparisons post. Values were considered significantly different when *P*<0.05 (95% CI). ^a^Significant difference from sham group, ^b^significant difference from RUP group, ^c^significant difference from OIR group

### Effect of RUP on ovarian inflammatory markers in female rats with OIR

**Fig. 1:**
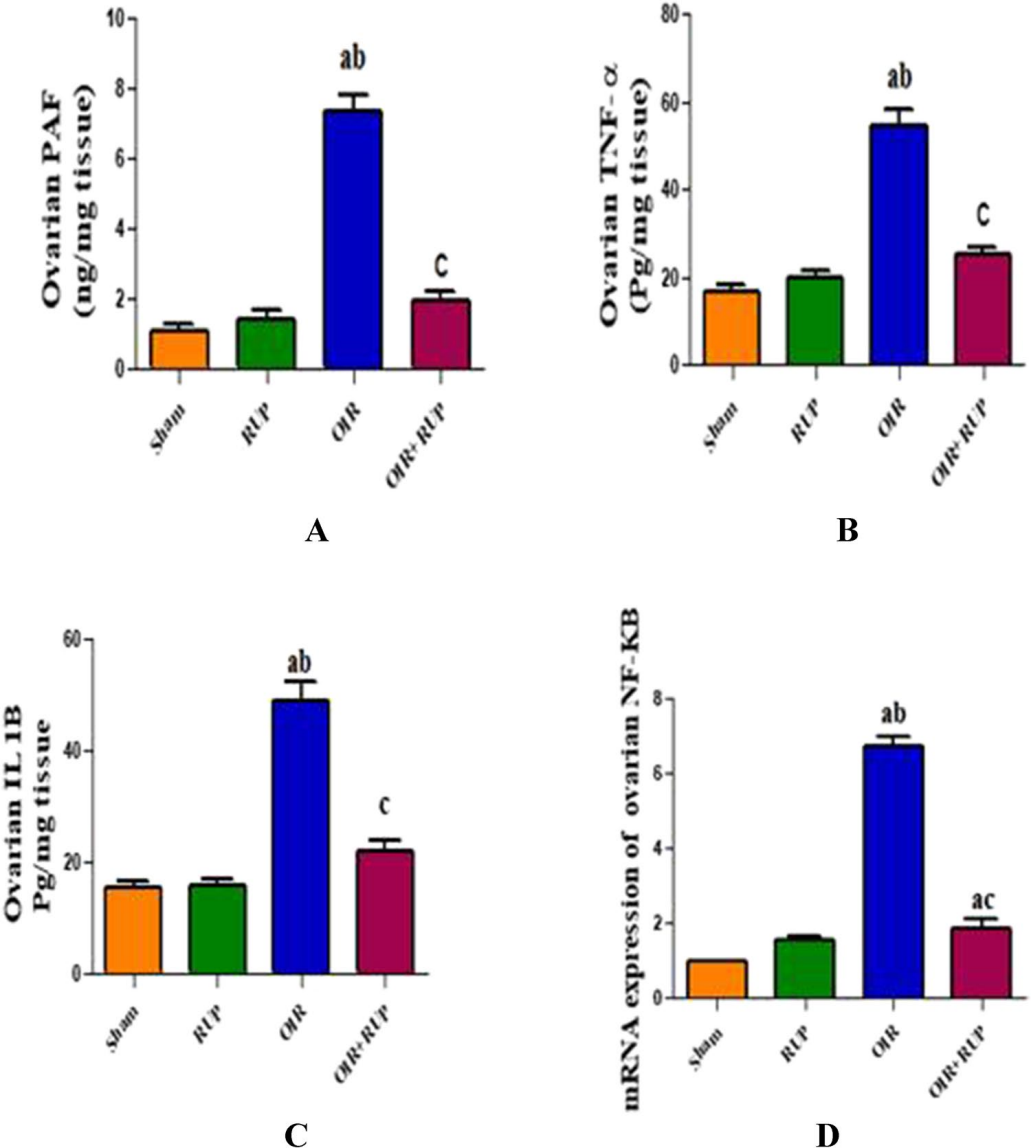
Effect of RUP on ovarian inflammatory markers in female rats with OIR: Data are expressed as mean ± SME (*n* = 8 rats/group). **a **Significant difference from sham group, **b** significant difference from RUP group,** c** significant difference from OIR group. RUP rupatadine, OIR ovarian ischemia reperfusion, TNF-α tumor necrosis factor alpha, IL-1β interleukin 1 beta, NFκB nuclear factor kappa-light-chain-enhancer of activated B cells, PAF platelet activating factor

OIR led to a significant increase in ovarian TNF-α, IL-1β, and PAF levels when compared with the sham and RUP groups. These alterations in levels of TNF-α, IL-1β, and PAF were significantly mitigated by pretreatment with RUP when compared to the OIR group. Also, ovarian mRNA expression of NF-κB was significantly increased in the OIR group when compared to the sham and RUP groups. Meanwhile, ovarian mRNA expression of NF-κB showed a significant decrease in the OIR + RUP group when compared to the OIR group (Fig. 1).

### Effect of RUP on ovarian HIF-1α in female rats with OIR

**Fig. 2:**
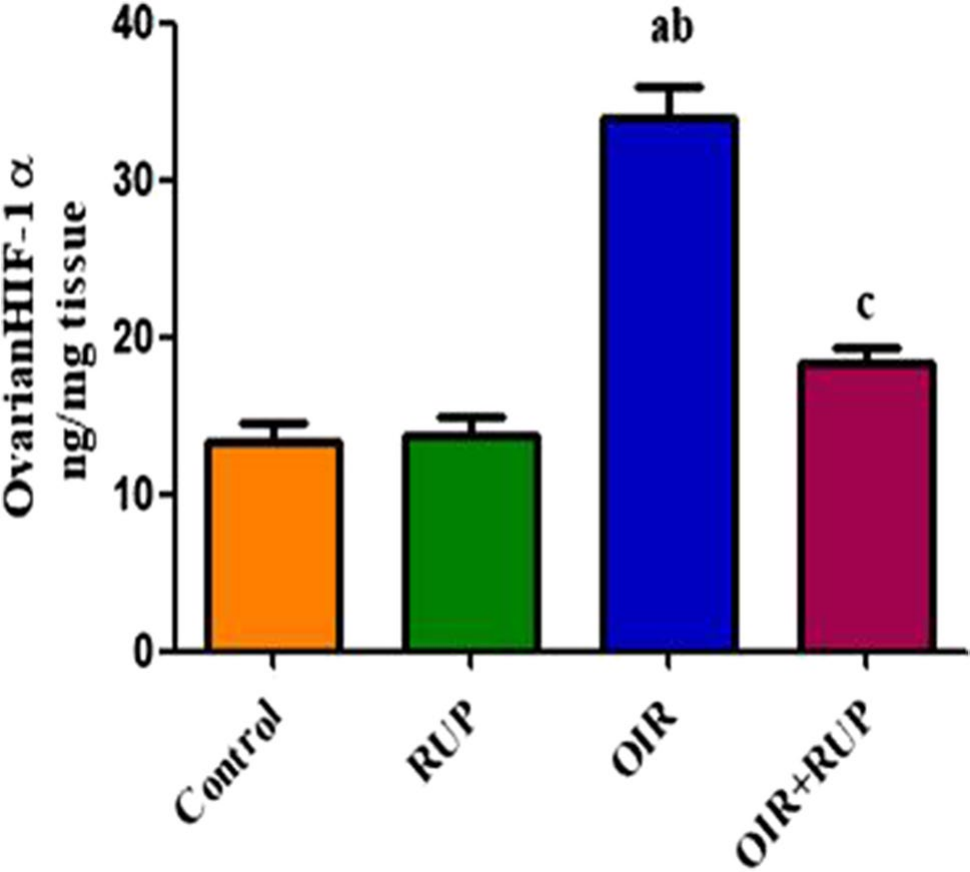
Effect of RUP on ovarian HIF-1α in female rats with OIR: Data are expressed as mean ± SME (*n* = 8 rats/group). Statistical analysis was performed using one-way ANOVA followed by Tukey’s multiple comparisons post. **a** Significant difference from sham group, **b** Significant difference from RUP group,** c** Significant difference from OIR group. RUP rupatadine, OIR ovarian ischemia reperfusion, HIF-1α hypoxia-inducible factor-1 alpha

As shown in Fig. 2, the OIR group showed a significant rise in the ovarian level of HIF-1α in comparison with the sham and RUP groups. Meanwhile, rats administered RUP before OIR had a significant reduction in the ovarian HIF-1α level as compared to the OIR group (Fig. 2).

### Effect of RUP on histopathological study of ovarian tissue

**Fig. 3:**
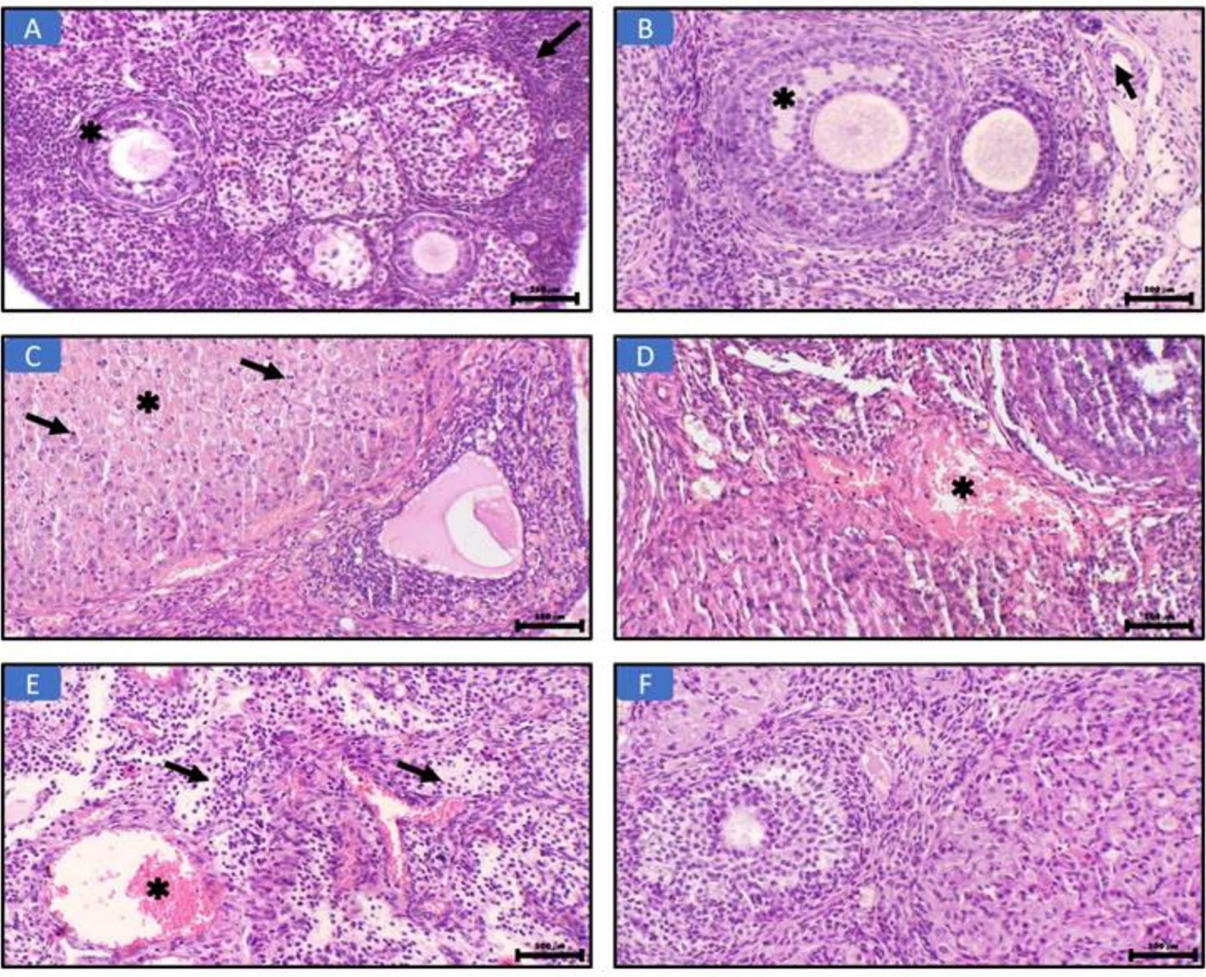
Effect of RUP on histopathological study of ovarian tissue: **A** Sham group, the normal ovarian tissue showing a follicle surrounded by granulosa cells (asterisk) and ovarian stroma (arrow) (H&E, × 200). **B** In the RUP group, the ovarian tissue shows a secondary follicle with oocyte (asterisk) and a normal blood vessel (arrow) (H&E, × 200). **C** OIR group, the ovary shows marked hemorrhage in the corpus luteum (asterisk) and inflammatory cellular infiltrate (arrows) (H&E, × 200). **D** In the I/R group, the ovary shows congestion (asterisk) (H&E, × 200). **E** In the OIR group, the ovary shows congestion (asterisk) and marked inflammatory cellular infiltrate (arrows) (H&E, × 200). **F** OIR + RUP group shows no detected pathological changes in the ovarian tissue (H&E, × 200)

Histological study (H&E, × 200) (Fig. 3) showed that the architecture of ovarian tissue was normal in the sham group (Fig. 3A), showing a follicle surrounded by granulosa cells (asterisk) and ovarian stroma (arrow). As shown in Fig. 3B in the RUP group**,** the ovarian tissue showed a secondary follicle with an oocyte (asterisk) and a normal blood vessel (arrow). Meanwhile, in the OIR group (Fig. 3C), the ovary shows marked hemorrhage in the corpus luteum (asterisk) and inflammatory cellular infiltrate (arrows). The ovary also showed congestion (asterisk) (Fig. 3D) and marked inflammatory cellular infiltrate (arrows) (Fig. 3E). On the other hand, the OIR + RUP group (Fig. 3F) showed no detected pathological changes in the ovarian tissue (H&E, × 200). These outcomes were consistent with the histological scoring improving (Table 3).

**Table 3:** Histopathological scoring for ovarian H&E in in female rats with OIR

Groups	Hemorrhage in the corpus luteum	Congestion in the ovarian stroma	Mononuclear cellular infiltrate
Sham	0	0	0
RUP	0	0	0
OIR	2.87 ± 0.13^ab^	2.5 ± 0.19^ab^	2.62 ± 0.18^ab^
OIR + RUP	0.38 ± 0.18^c^	0.50 ± 0.22^c^	0.50 ± 0.18^c^

*RUP* rupatadine, *OIR* ovarian ischemia reperfusion

Data are expressed as mean ± SEM. (*n* = 8 rats/group). Statistical analysis was performed using one-way ANOVA followed by Tukey’s multiple comparisons post. Values were considered significantly different when *P*<0.05 (95% CI). ^a^Significant difference from sham group, ^b^significant difference from RUP group, ^c^significant difference from OIR group

### Effect of RUP on immunohistochemical expression of VEGF and caspase 3 in ovarian tissue

**Fig. 4:**
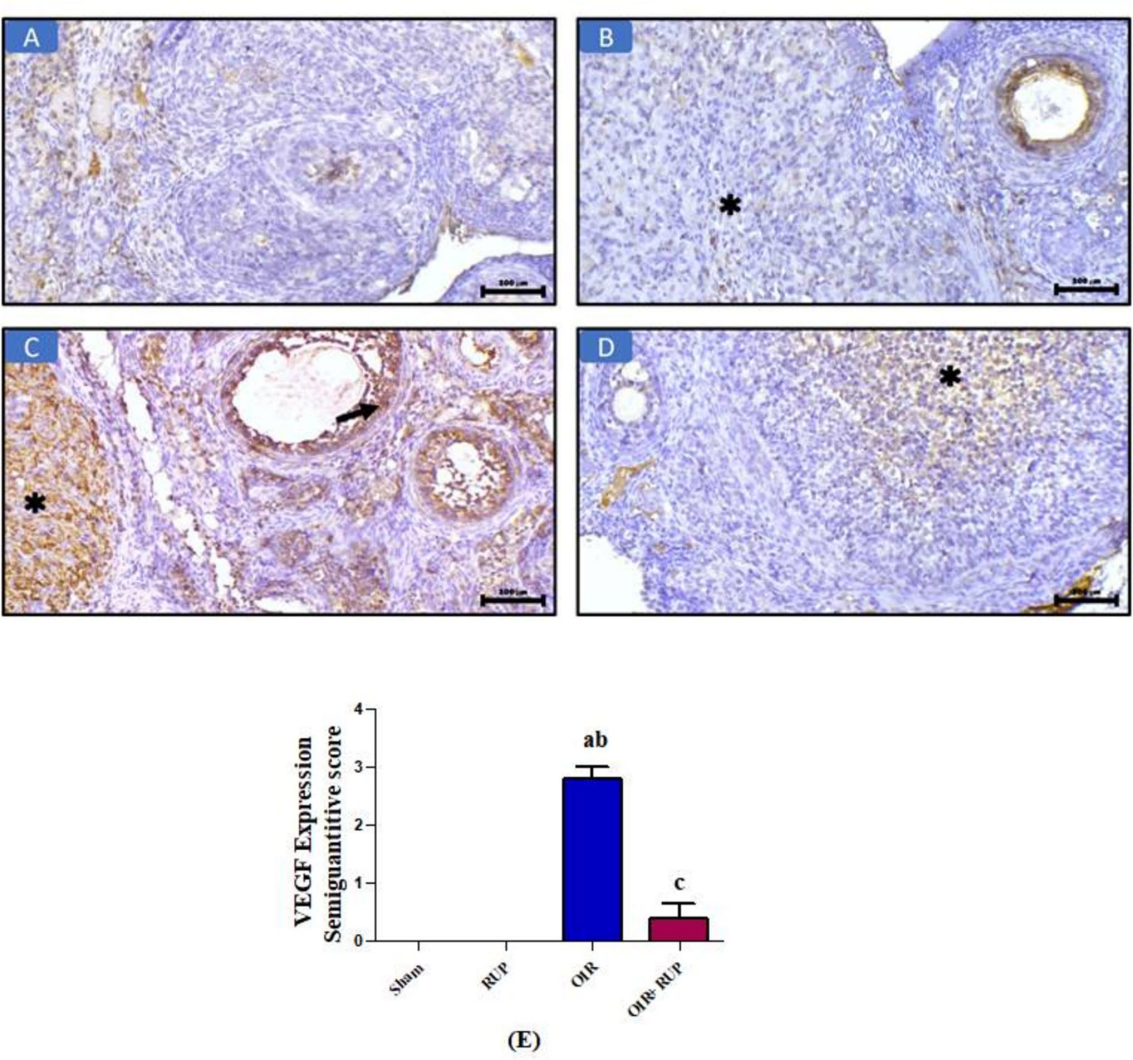
Effect of RUP on immunohistochemical expression of VEGF: **A** VEGF expression in the sham group: the picture demonstrates negative expression in ovarian tissue (IHC, × 200). **B **VEGF expression in the RUP group: the picture demonstrates negative expression in ovarian stroma and corpus luteum (asterisk) (IHC, × 200). **C** VEGF expression in the OIR group: the picture demonstrates diffuse cytoplasmic expression in the corpus luteum (asterisk) and follicular cells (arrow) (IHC, × 200). **D** VEGF expression in the OIR + RUP group: the picture demonstrates scattered focal cytoplasmic expression (asterisk) (IHC, × 200). **E** Data are expressed as mean ± SME (*n* = 8 rats/group). Values were considered significantly different when *P* < 0.05. **a** Significant difference from sham group, **b** significant difference from RUP group,** c** significant difference from OIR group. RUP rupatadine, OIR ovarian ischemia reperfusion

Immunohistochemical analysis of VEGF (IHC, × 200) (Fig. 4) exhibited no expression of VEGF in ovarian tissue in both sham and RUP groups (Fig. 4A & B) in ovarian stroma and corpus luteum (asterisk). However, in the OIR group (Fig. 4C), the picture demonstrated diffuse cytoplasmic expression of VEGF in the corpus luteum (asterisk) and follicular cells (arrow). On the other hand, the OIR + RUP group (Fig. 4D) revealed scattered focal cytoplasmic expression of VEGF (asterisk) (IHC, × 200). Semiquantitative scoring: Data represent the mean ± SEM. A *p*-value < 0.05 is set for significance (Fig. 4E).

**Fig. 5:**
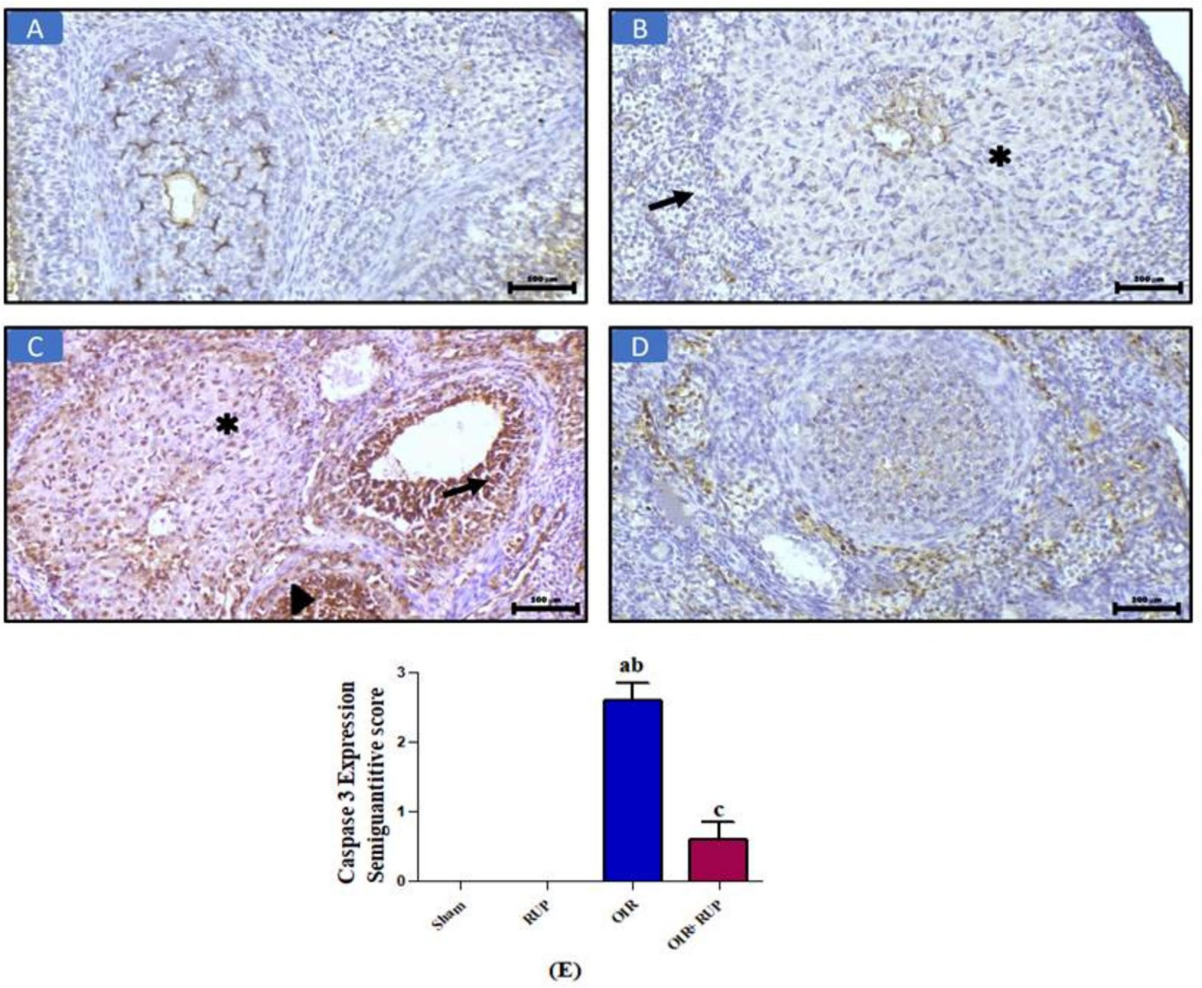
Effect of RUP on immunohistochemical expression of caspase 3: **A** Caspase 3 expression in the sham group: the picture demonstrates negative expression in ovarian tissue (IHC, × 200). **B** Caspase 3 expression in the RUP group: the picture demonstrates negative expression in the corpus luteum (asterisk) and ovarian stroma (arrow) (IHC, × 200). **C** Caspase 3 expression in the OIR group: the picture demonstrates diffuse nuclear expression in the corpus luteum (asterisk), follicular cells (arrow), and granulosa cells (arrowhead) (IHC, × 200). **D** Caspase 3 expression in the OIR + RUP group: the picture demonstrates scattered focal nuclear expression. **E** Data are expressed as mean ± SME (*n* = 8 rats/group). Values were considered significantly different when *P* < 0.05. **a** Significant difference from sham group, **b** significant difference from RUP group, **c** significant difference from OIR group. RUP rupatadine, OIR ovarian ischemia reperfusion

Similarly, immunohistopathological analysis of caspase 3 (IHC, × 200) (Fig. 5) revealed a negative expression of caspase 3 in ovarian tissue in both the sham and RUP groups in the corpus luteum (asterisk) and ovarian stroma (arrow) (Fig. 5A & B). On the other hand, caspase 3 expression in the OIR group (Fig. 5C) demonstrated diffuse nuclear expression in the corpus luteum (asterisk), follicular cells (arrow), and granulosa cells (arrowhead). In contrast, in the OIR + RUP group, the picture demonstrated scattered focal nuclear expression (Fig. 5D). Semiquantitative scoring: Data represent the mean ± SEM. A *p*-value < 0.05 is set for significance (Fig. 5E).

## Discussion (Fig. 6)

**Fig. 6:**
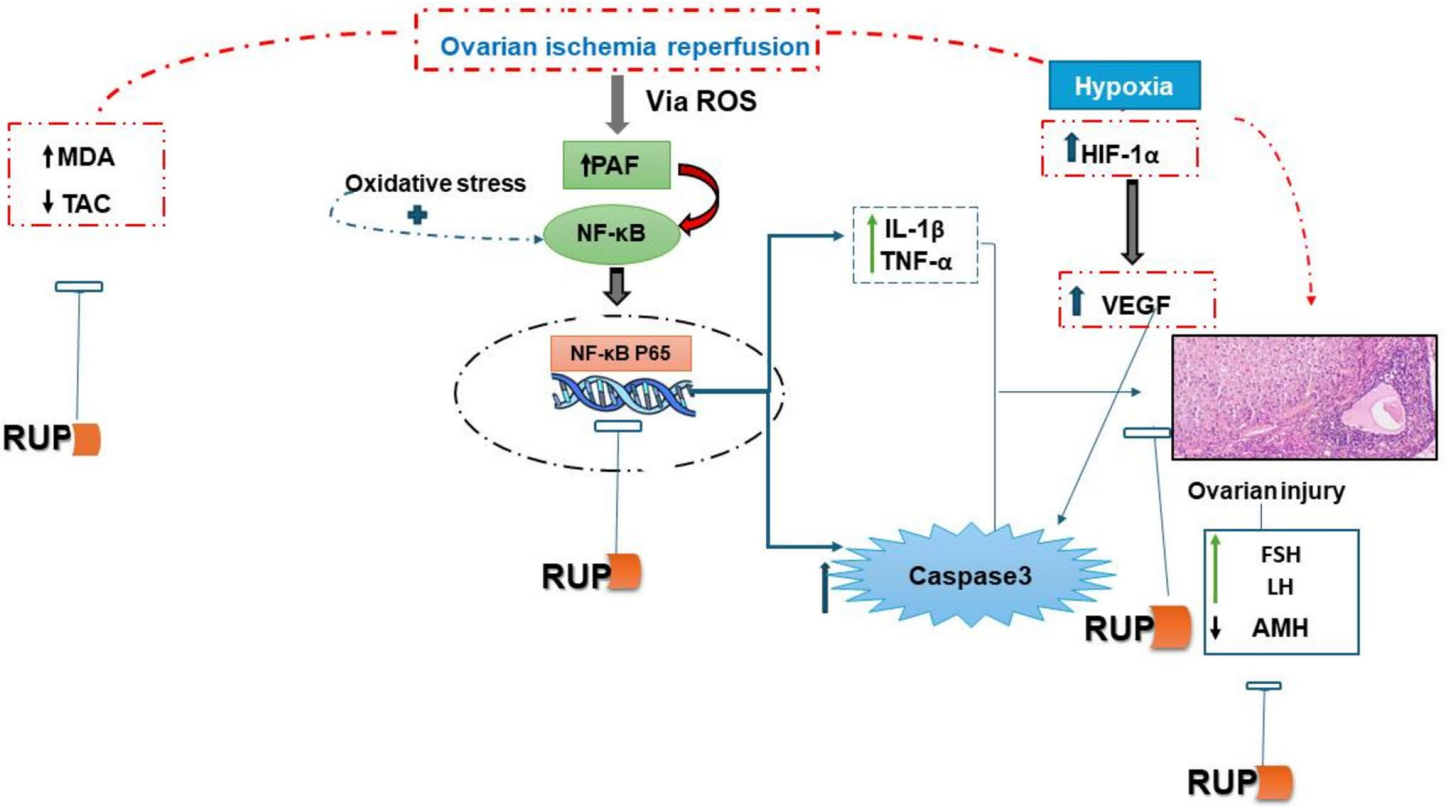
Schematic diagram showing the proposed molecular mechanisms explaining the protective effects of rupatadine in ovarian ischemia reperfusion in rats. RUP rupatadine, ROS reactive oxygen species, TNF-α tumor necrosis factor alpha, IL-1β interleukin 1 beta, NFκB nuclear factor kappa-light-chain-enhancer of activated B cells, PAF platelet-activating factor, HIF-1α hypoxia-inducible factor-1 alpha, VEGF vascular endothelial growth factor

OIR injury remains a critical gynecological concern, often arising from ovarian torsion due to adnexal masses or surgical manipulation. Although detorsion restores blood flow, the subsequent reperfusion can paradoxically exacerbate tissue injury through oxidative stress and inflammation. As highlighted by Abdel-Gaber et al. (2020), conservative management is sometimes considered to preserve ovarian function in such cases.

In the present study, experimental induction of OIR resulted in significant structural and functional ovarian impairment, as evidenced by histopathological alterations and hormonal disturbances. Compared to sham-operated controls, OIR rats exhibited decreased serum levels of AMH alongside elevated FSH and LH levels. AMH, produced by granulosa cells of growing follicles, is a reliable marker of ovarian reserve (Eken et al. 2019). The observed decline in AMH, coupled with elevated gonadotropins, suggests compromised follicular development and disrupted hypothalamic-pituitary-ovarian axis feedback findings consistent with those of Bagheri et al. (2023).

Notably, treatment with RUP ameliorated these hormonal disturbances. This regulatory effect is likely attributable to RUP’s pleiotropic pharmacological actions, particularly its anti-inflammatory, antioxidant, and anti-apoptotic properties. Histopathological analyses further supported RUP’s protective role, revealing preserved ovarian architecture in pretreated animals.

The mechanisms underlying IR-induced damage are multifaceted. Key pathological features include oxidative stress, endothelial dysfunction, neutrophil infiltration, and the release of pro-inflammatory cytokines. The reperfusion phase, in particular, introduces a surge of ROS, leading to lipid peroxidation, mitochondrial dysfunction, and cellular injury (Şimşek et al. 2023). This study demonstrated increased MDA levels and decreased TAC in the OIR group, indicating oxidative imbalance—findings supported by Ileriturk et al. (2024)**.** RUP pretreatment effectively restored TAC and reduced MDA, aligning with its documented antioxidant activity in various preclinical models (Ahmed et al. 2021; Ibrahim et al. 2022; Khalaf et al. 2022; Abdel-Aziz et al. 2024; Tangul et al. 2025).

In parallel with oxidative stress, inflammation is a major driver of reperfusion injury. Elevated levels of NF-κB, PAF, TNF-α and IL-1β were observed in the OIR group, confirming a robust inflammatory response. PAF plays a central role in leukocyte activation and is implicated in ovarian inflammatory pathology (Ibrahim et al. 2022; Kölükçü et al. 2024; Öztürk et al. 2025). NF-κB, activated by both ROS and PAF, orchestrates the expression of multiple pro-inflammatory genes (Piechota-Polanczyk and Fichna 2014; Yuksel et al. 2023; Abdelzaher et al. 2024; Güvenç et al. 2024). Interestingly, RUP exhibited a protective effect against inflammation; it significantly attenuated these markers, confirming its anti-inflammatory efficacy. These findings are consistent with prior studies where RUP conferred protection in testicular IR injury (Abdel-Aziz et al. 2024) and diabetic nephropathy (Hafez et al. 2020). Moreover, RUP’s ability to block PAF-mediated mast cell activation, as shown by Demopoulos et al. (2020), further supports its therapeutic versatility in inflammatory conditions.

The pathophysiology of OIR also involves hypoxia-induced signaling and apoptotic pathways. Elevated expression of HIF-1α, VEGF, and caspase-3 was detected in the OIR group, reflecting tissue hypoxia, compensatory angiogenesis, and cell death (Demir et al. 2021; Dong et al. 2021). While HIF-1α can mediate protective effects by upregulating VEGF during ischemia (Zhu et al. 2014; Soares et al. 2019), persistent overexpression may contribute to pathological angiogenesis and fibrosis (Canillioglu and Senturk 2020; Deger and Cavus 2020; Toprak and Deveci 2023). Similarly, caspase-3 is a central effector of apoptosis and is activated by oxidative stress. RUP treatment significantly reduced the immunoexpression of HIF-1α, VEGF, and caspase-3, indicating suppression of hypoxia-related damage and apoptosis (Jin et al. 2001). These findings are in agreement with previous reports demonstrating RUP-mediated downregulation of the HIF-1α/VEGF axis in ulcerative colitis (Ibrahim et al. 2022) and liver fibrosis (Didamoony et al. 2023).

It is worthy to note that the result of the current study is limited, as it lacks evaluation of RUP in different doses and in a curative model. Therefore, we strongly recommend further studies for evaluation of such limited points.

## Conclusion

Collectively, the current findings underscore the protective potential of RUP against OIR-induced structural and functional ovarian injury. Through its antioxidant, anti-inflammatory, and anti-apoptotic effects, RUP helped preserve hormonal balance, reduced oxidative and inflammatory markers, and maintained ovarian tissue integrity. These results support the potential repurposing of RUP as an adjunctive therapy in the clinical management of ovarian torsion and IR injuries.

## Data Availability

All source data for this work (or generated in this study) are available upon reasonable request.
